# Syphilis: A Forgotten Sexually Transmitted Disease?

**DOI:** 10.3389/fpubh.2014.00015

**Published:** 2014-03-06

**Authors:** Amy Burnett, Hatim A. Omar

**Affiliations:** ^1^Division of Adolescent Medicine, Kentucky Children’s Hospital, University of Kentucky College of Medicine, Lexington, KY, USA

**Keywords:** syphilis, STD, adolescent sexuality, secondary syphilis

## Introduction

Syphilis has declined significantly over the last 30 years. Due to that success, many physicians no longer have the skills to recognize its symptoms (1). We present a case of syphilis as a demonstration of this fact and hope that health professionals in the field of adolescent medicine will think about this diagnosis in their clinical work.

## Case Report

A 17-year-old African-American male presented to the adolescent medicine clinic with the complaint of bilateral inguinal swelling for 3 weeks. Patient denied pain to inguinal area, testicular pain, penile discharge, dysuria, abdominal pain, fever, weight loss, or fatigue. The patient denied history of inguinal hernias or any recent heavy lifting or straining. The only other complaint at the time of visit was a rash to the right chin, right torso, right forearm, and genital area for several days. The patient was seen in an emergency room and the rash diagnosed as contact dermatitis that was due to a change in soap 2 days prior to the rash appearing.

Patient was preparing to enter his senior year of high school. He denied tobacco, alcohol, or drug use. The patient had been sexually active and had four lifetime partners, all females. Patient stated he did use condoms with all sexual intercourse. He did have multiple tattoos and denied any international travel.

**Figure 1:**
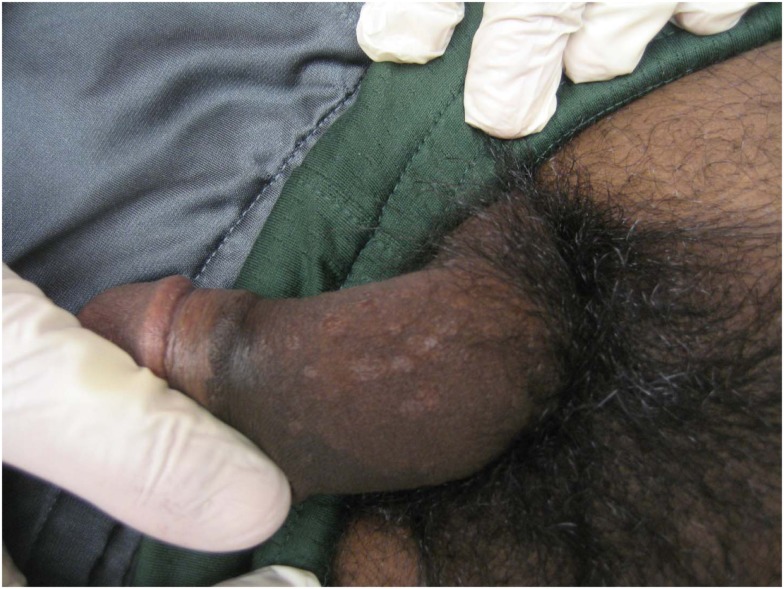
**Rash associated with secondary syphilis resembling allergic reaction**.

On exam, this patient was noted to have a hypopigmented, raised flat rash to the right lower face and chin, right forearm, right torso, penis, and scrotum (see Figure 1). Lesions were also found on palms and soles. The rash was non-tender and no erythema was noted. The patient also had cervical lymphadenopathy to the left lateral neck with mild tenderness and bilateral palpable axillary nodes that were non-tender. The most significant finding was bilateral 3 cm × 2.5 cm inguinal nodes that were non-tender.

After extensive testing was performed to rule out infectious process, the patient had a positive rapid plasma regain (RPR) and a positive follow-up *Treponema pallidum* particle agglutination assay (TP-PA), which confirmed the diagnosis of secondary syphilis and appropriate treatment was administered. The patient denied noticing an initial chancre associated with primary syphilis.

## Discussion

Due to the presentation of this patient, it would be very easy to misdiagnose the symptoms as viral illness and/or dermatitis. The lowest documented rates of syphilis were in the year 2000, but have been slowly increasing over the past decade (1). It is important to consider this diagnosis in all individuals with any known high-risk behaviors and screen at any appropriate opportunity (1–5). It is impossible to verify if this patient contracted the infection from sexual activity or the tattooing process. Remembering to consider syphilis in sexually active adolescents presenting with unusual rash is key to early diagnosis.

## References

[1] Sexually Transmitted Disease Surveillance. Centers for Disease Control (2011). Available from: http://www.cdc.gov/std/stats11/syphilis.htm

[2] Workowski KA Berman S Centers for Disease Control and Prevention (CDC) Sexually transmitted diseases treatment guidelines, 2010. MMWR Recomm Rep (2010) 59(RR-12):1–110 21160459

[3] Sexually Transmitted Diseases Treatment Guidelines. (2010). Available from: http://www.cdc.gov/std/treatment/2010/21160459

[4] Syphilis, CDC Fact Sheet. (2013). Available from: http://www.cdc.gov/std/syphilis/STDFact-Syphilis.htm

[5] Syphilis. (2013). Available from: http://www.cdc.gov/std/syphilis/default.htm

